# Examining the association between opium use, cigarette smoking and alcohol consumption with the liver enzyme levels in a population-based study: Fasa Persian cohort data

**DOI:** 10.1186/s13104-021-05891-3

**Published:** 2022-01-05

**Authors:** Mostafa Bijani, Azizallah Dehghan, Saeed Razavi, Shahnaz Karimi

**Affiliations:** 1 Department of Medical Surgical Nursing, Fasa University of Medical Sciences, Fasa, Iran; 2 Noncommunicable Diseases Research Center, Fasa University of Medical Sciences, Fasa, Iran; 3 Department of Medical Surgical Nursing, School of Nursing, Fasa University of Medical Sciences, Fasa, Iran

**Keywords:** Smoking, Alcohol consumption, Opium, Liver function tests, Cohort study

## Abstract

**Objectives:**

Opium use, cigarette smoking, and alcohol consumption are serious health problems in many countries including Iran. The present study aimed to examine the association between the opium use, cigarette smoking and alcohol consumption with liver enzyme levels in Southern Iran. This analytical cross-sectional study was conducted in 2020. The entire population of the Fasa Persian cohort study in the southern region of Iran was selected as the sample. Accordingly, 10,145 people participated in the study.

**Results:**

Results indicated that there was a significant relationship between cigarette smoking and alcohol consumption with liver enzymes (AST, ALT, and ALP). There was also a significant relationship between inhaled opium and liver enzymes, but oral opium revealed no significant relationship with the activity of liver enzymes. Accordingly, policymakers of the health care system are recommended to hold educational programs to improve the health literacy level of the society and take effective preventative strategies in reducing the use of these substances.

## Introduction

Opium use, cigarette smoking, and alcohol consumption are serious health challenges in most countries worldwide. Alcohol consumption is one of the leading risk factors for the burden of disease globally and has greatly reduced the level of public health in the world [1]. According to the World Health Organization, the annual global average alcohol consumption is 8 L per person aged 15 years and over in the United States, while the European countries consume the most alcohol in the world (11.3 L per person) [2]. In Iran, the production and consumption of alcoholic beverages are prohibited, so they are produced or consumed secretly; thus, it is too difficult to measure the exact amount of the alcohol consumed. According to the World Health Organization’s report (2018), Iran ranked ninth out of 189 countries in terms of per capita alcohol consumption. This report also indicates that every Iranian person aged 15 or older who “drinks alcohol regularly” consumes an average of 28.4 L of pure alcohol in 2016, while they consumed 24.8 L of pure alcohol in 2010 [3].

The alcohol-specific death rate is another challenge to the health care system. More than 2,90,000 people have passed away due to excessive alcohol use and a total of 7.6 million years of life have been lost due to disability and premature death in 2016. In this year, alcohol abuse was also identified as the seventh leading risk factor for death and disability [4].

Drug addiction is another major challenge to the health care system. Unfortunately, drug use is increasing in the world, Iran included. The severity and complexity of the world drug situation are aggravating. World Drug Report [5] announced that 35 million people worldwide suffered from drug use disorders. It also indicated that addiction would have been exploded like a bomb by 2025.

According to the United Nations’ report, 4,50,000 people pass away due to drug abuse every year worldwide [6]. There is little information available on the prevalence of drug use and addiction in Iran. Nevertheless, Iran Drug Control Headquarters reported that there were about 1.2 million addicts in Iran in the years 2012 and 2013 [7]. They also reported that this number reached 2.8 million people in 2019, which is equivalent to 5% of the population of Iran, indicating a rising trend in the rate of addiction in Iran. Further, about 4000 people pass away due to drug use in Iran every year, and it can be due to its proximity to its neighboring country, Afghanistan, which is the largest producer of opium in the world [8].

Drug addiction causes pain and suffering for the addicts, their family, as well as their body organs. For example, alcohol consumption and drug abuse directly influences the liver as the hepatocytes are responsible for drug metabolism [9]. Some studies have found that alcohol consumption and drug abuse cause various liver diseases such as fatty liver disease, hepatitis, and liver failure [10, 11]. Increased serum enzyme level is one of the most common symptoms of liver damage [12].

Marks et al. [13] conducted a study on heroin and cocaine users and found that the level of alkaline phosphatase increased slightly above normal in their bodies. They also showed that most addicts had damaged livers. Stenger et al. [14] also showed that liver diseases were more prevalent in people who consumed alcohol and psychedelic drugs; they indicated that the level of alkaline phosphatase increased with the consumption of alcohol and other substances. Cheung et al. [15] conducted a study at the American Institute of Healthcare and Fitness from 1999 to 2004 and reported that cigarette smoking plus non-alcoholic beverage consumption elevated the serum ALP levels. Nevertheless, Pedrazzoni et al. [16] carried out a study on 130 male heroin addicts and showed that the ALP level in the experimental group was not much different from that in the control group. Moreno et al.’s [17] study suggested that alcohol consumers experienced lower ALP levels, but higher AST, GGT, and bilirubin levels compared to those who did not consume alcohol. Thus, since cigarette smoking, drug use, and alcohol consumption can cause liver disorders and greatly influence the level of liver enzymes, the present study aimed to examine the association between cigarette smoking, drug use and alcohol consumption with the levels of ALT, AST, and ALP in Persian Fasa cohort study in Southern Iran.

## Main text

### Methods

This is an analytical cross-sectional study. The study population consisted of the individuals covered by Fasa Cohort in the south of Iran. The data used in this research were driven from the baseline survey of the PERSIAN (Prospective Epidemiological Research Study in Iran) Cohort Study (Fasa non-communicable disease cohort study). In this study, a population-based cohort of 10,000 individuals aged 35–70 years old from Sheshdeh, a suburb of Fasa city and its 24 surrounding was enrolled. Detailed demographic, socioeconomic, anthropometric, nutritional, and medical data were obtained for each participant; a health care worker and physician helped us to do limited physical examinations and determined their level of physical activity. Routine laboratory assessments were done and a comprehensive biobank was compiled for upcoming biological investigations. All data were stored online through dedicated software [18]. The entire population of the Fasa Persian cohort study was selected as the sample. It was conducted through census. Fasa Persian cohort is a longitudinal study performed on more than 10,000 people aged 35–70 years old in Sheshdeh and Qarebolagh District of Fasa, Fars. In the present study, 11,454 individuals were invited to take part in the study, but 453 people were excluded as they had handed out incomplete questionnaires. Further, 856 people who were under 35 years old were excluded from the study. Finally, 10,145 people were selected as the study sample.

As mentioned above, sampling was done in a full-census manner; all the information recorded in the Fasa Persian cohort study, Sheshdeh branch, Fars province in Southern Iran was fully analyzed.

The instrument used for collecting the data was a questionnaire containing three information sections. The first part was on demographic information including some items such as age, gender, and marital status.

In the second part, some information about direct and indirect cigarette smoking, opium abuse in the form of inhaled and oral opium, and alcohol consumption was collected. In the last section, some questions about liver function tests including alkaline phosphatase (ALP), alanine aminotransferase (ALT), aspartate aminotransferase (AST) were asked and recorded. The normal range of liver tests is as follows: ALT: 29–33 U/L, AST: 10–40 U/L, and ALP: 45–115 U/L. The sampling was done by four laboratory technicians and a lab supervisor who worked in the laboratory. After taking samples from participants in the field, they were transferred into cool boxes to the laboratory for processing. Different laboratory data were provided for each participant. These include ALT, AST, and ALP. We recorded the laboratory data in an electronic database. These data were then entered into the main database which included all participants’ detailed information recorded through the questionnaires.

Inclusion criteria were age over 35 years and fully completed self-reported questionnaire. On the other hand, exclusion criteria were incomplete questionnaires and suffering from liver disease, osteoporosis, cancer, and hepatitis.

### Ethical considerations

All participants gave written informed consent to participate in the study. The present study was conducted in accordance with the principles of the revised Declaration of Helsinki, a statement of ethical principles which directs physicians and other participants in medical research involving human subjects. The participants were assured of their anonymity and confidentiality of their information. Moreover, the study was approved by the local Ethics Committee of Fasa University of Medical Sciences, Fasa, Iran (Ethical code: IR.FUMS.REC.1398.039).

Data were analyzed by SPSS software version 22 using descriptive and analytical statistics such as independent sample t test, chi-squared test, one-way analysis of variance (one-way ANOVA) and linear regression (forward method). The P value  < 0.050 was considered as the significant level (P  < 0.05).

### Results

This study was conducted on 10,145 subjects, with a mean age of 48.64 ± 9.58 years old. Based on the results, 4580 (45.2%) subjects were male and 5557 (54.8%) female. The subjects’ maximum age was 87 years and the minimum 35 years old. 1946 (19.8%) participants were current smokers and 2239 (22.1%) were opium users (Table 1). Approximately, 224 (2.2%) of the cohort population consumed alcohol regularly; they were mostly male and younger compared to those who did not consume alcohol. The activity of liver enzymes of alcohol consumers and non-consumers was compared, and alcohol consumers had significantly lower ALP (P  = 0.016), higher AST (P  < 0.001), and higher ALT (P  < 0.001). The activity of liver enzymes of smoking and non-smoking participants was compared, showing that smokers had significantly lower ALT (P  < 0.001), higher AST (P  = 0.0.002), and higher ALP (P  < 0.001). There was a significant relationship between the use of inhaled opium with ALT (P  = 0.010) and ALP (P  = 0.005), but the use of oral opium showed no significant relationship with the activity of liver enzymes. There was a significant relationship between gender and liver enzymes, ALT (P  < 0.001) and AST (P  < 0.001), but there was no significant relationship between gender and ALP (P  = 0.087) (Table 2). Pearson’s correlation coefficient revealed that ALP (r  = 0.219, P   < 0.001) and AST(r  = 0.019, P  = 0.61) had a direct relationship with age, but an inverse association with ALT (r  = − 0.064, P  < 0.001). In a linear regression model, we added all the variables which were correlated with the activity of liver enzymes. Linear regression test indicated that age (P  < 0.0001) and use of oral opium (P  = 0.002) had a significant relationship with ALP. Multivariate analysis showed that age (P  < 0.0001), gender (P  < 0.0001), inhaler opium (P  < 0.0001), oral opium (P  = 0.033), and alcohol consumption (P  = 0.033) were significantly related to ALT; finally, gender (P  < 0.0001) and inhaler opium (P  = 0.006) showed a significant association with AST (Table 3). Cut-off levels enzyme showed that 326 (2.3%) of the participants had AST enzyme levels higher than normal. 1498(14.8%) of the participants had ALT enzyme levels higher than normal. Also 9734(96%) of the participants had ALP enzyme levels higher than normal.

**Table 1:** Participants’ demographic information

Variable		Frequency	Percentage
Gender	Male	4582	45.2
	Female	5563	54.8
Marital status	Single	375	3.7
	Married	9022	88.9
	Widow/widower	644	6.3
	Divorced	104	1.1
Inhaled opium	Yes	2196	21.7
	No	7949	78.3
Oral opium	Yes	87	8
	No	10,058	99.2
Smoking	Yes	1946	19.18
	No	8199	80.81
Alcohol consumption	Yes	224	2.2
	No	9921	97.8

**Table 2:** The association between demographic variables, cigarette Smoking, opium use, alcohol consumption, and liver enzymes

Variable		AST	P value	ALT	P value	ALP	P value
Gender	Male	23.80 ± 9.09	< 0.001^a^	25.92 ± 16.63	< 0.001^a^	210.98 ± 68.23	0.087^a^
	Female	21.55 ± 7.83		21.40 ± 11.91		208.52 ± 74.28	
Marital status	Single	22.00 ± 6.87	0.001^b^	22.49 ± 14.40	< 0.001^b^	197.87 ± 59.17	< 0.001^b^
	Married	22.65 ± 8.59		23.64 ± 14.59		209.01 ± 72.27	
	Widow/widower	22.19 ± 8.39		22.01 ± 12.13		229.36 ± 68.14	
	Divorced	19.59 ± 4.74		18.16 ± 9.29		183.84 ± 48.26	
Smoking	No	22.51 ± 8.09	0.002^a^	23.60 ± 14.03	< 0.001^a^	208.07 ± 72.98	< 0.001^a^
	Yes	22.79 ± 10.02		22.78 ± 15.95		216.19 ± 65.20	
Inhaled opium	No	22.35 ± 8.25	0.290^a^	23.30 ± 13.65	0.010^a^	209.37 ± 71.61	0.005^a^
	Yes	23.35 ± 9.31		23.94 ± 16.87		210.59 ± 71.66	
Alcohol consumption	No	22.53 ± 8.41	< 0.001^a^	23.36 ± 14.18	< 0.001^a^	209.69 ± 71.99	0.016^a^
	Yes	24.42 ± 11.97		27.33 ± 22.51		206.68 ± 50.66	
Oral opium	No	22.56 ± 8.50	0.514^a^	23.45 ± 14.42	0.834^a^	209.43 ± 71.57	0.110^a^
	Yes	23.57 ± 7.95		22.53 ± 12.94		233.88 ± 73.53	

^a^Independent sample t test

^b^One way analysis of variance

**Table 3:** The association between age, gender, opium use, alcohol consumption, and liver enzymes

Dependent variable	Independent variable	B	Std. error	t	P value
ALP	Age	1.438	0.074	21.295	< 0.0001
	Oral opium	22.15	6.38	3.021	0.001
ALT	Gender	− 6.080	0.243	− 17.905	< 0.0001
	Inhaled opium	− 3.566	0.418	− 8.652	< 0.0001
	Age	− 0.102	0.015	− 6.880	< 0.0001
	Oral opium	− 3.425	1.678	− 2.137	0.036
	Alcohol consumption	2.182	1.021	2.251	0.024
	Oral opium and alcohol consumption	3.84	0.89	4.139	0.004
AST	Gender	− 2.294	0.30.2	− 10.309	< 0.0001
	Inhaled opium	− 0.528	0.224	− 2.629	0.005
	Inhaled opium + alcohol consumption	2.032	0.98	2.67	0.042

Linear regression (forward method)

### Discussion

The prevalence of alcohol consumption, opium use, and cigarette smoking as well as their side effects has always been one of the serious challenges to the health care system in most countries [19]. Several studies have shown detrimental effects of alcohol consumption, opium use, and cigarette smoking on the body organs, such as liver damage [20, 21].

The current study aimed to examine the population of the Fasa Persian cohort study in the southern region of Iran to show the association between cigarette smoking, opium use, as well as alcohol consumption, with liver enzyme levels (AST, ALT, and ALP). It also explored some variables such as active and inactive cigarette smoking, marital status, age, oral and inhaled opium use, and alcohol consumption plus their effect on liver enzymes. Linear regression test revealed that age and oral opium had a significant correlation with ALP; also, age, gender, inhaled opium, and oral opium showed a significant correlation with ALT. Finally, gender and inhaled opium indicated a significant correlation with AST.

Nivukoski et al. [22] conducted a study in Finland, reporting that alcohol consumption and cigarette smoking played a key role in the development of liver disease, such as non-alcoholic fatty liver. They concluded that policymakers and managers of the health care system need to hold educational programs to improve the health literacy levels of the society and take effective preventative measures in reducing the consumption of these substances.

Consumption of alcohol in Iran is prohibited by law, but people usually honestly report alcohol or drug use when asked by the treatment team personnel. Thus, the treatment team can play a significant role in clarifying the risks of opium and alcohol consumption.

Samejo et al. [23] carried out a study in Pakistan and indicated that alcohol consumption elevated the levels of ALP, AST, and ALT. They also showed that there was a significant relationship among age, gender, and liver enzymes, which is consistent with the results of the present study. Khalili et al. [24] revealed that opium consumption increased both the risk of cardiovascular disease, stroke and lung cancer and the levels of liver enzymes such as ALP, AST, and ALT. Masoomi et al. [25] indicated that cigarette smoking and inhaled opium triggered inflammatory reactions in the liver and prepared the grounds for acute liver damage. Pawan et al. [26] studied the damaging effects of opium use on the liver and lungs in chronic opium addicts in Rajasthan, India, and indicated that long-term and chronic opium use raised the risk of liver and lung damage. Elsewhere, Jerkeman et al. [27] carried out a study on death from liver disease in a cohort of injecting opioid users in a Swedish city; they reported that the death rate significantly increased in these people. Thus, seemingly, the inhaled opium brings about inflammatory reactions in the liver more than its oral consumption.

Addolorato et al. [28] carried out a study in Italy and showed that alcohol consumption elevated the level of liver enzymes and the likelihood of liver cirrhosis and liver cancer. This study was conducted only in the southern region of Iran. Since opium use, cigarette smoking, and alcohol consumption are affected by various factors, including cultural, economic, and social conditions; therefore, the results cannot be generalized, so it is necessary to conduct the same study in other parts of Iran or even in other countries.

### Conclusion

This study reported the effect of opium use, cigarette smoking, and alcohol consumption on the level of liver enzymes. People use cigarettes, opium, and alcohol to reduce the stresses of everyday life. They use opium not only as a habit, but also based on many false beliefs about the positive effects of opium on fatty liver and other diseases such as heart disease, and diabetes. Based on the findings of the present study, consuming opium and drinking alcohol increase the liver function tests related to accelerated liver diseases and the risk of liver cancer and mortality. In this study, the liver enzyme levels were similar in smokers and inhaled opium users; this should be taken into account. It is necessary to take preventative measures to stop liver damage in people who are addicted to alcohol, drugs, and smoking. Due to the prevalence of consumption and misconception, policymakers and managers of the health care system are recommended to hold educational programs to improve the health literacy levels of society.

### Limitations

According to the fact that opium use, cigarette smoking, and alcohol use are affected by cultural, social, and economic factors, the results of this study cannot be generalized to other parts of Iran and other countries, so similar studies are suggested to be conducted in other parts of Iran as well as in other countries. In our study, those who consumed medications which affect liver function test (for example: antiepileptic drugs. Cholesterol-lowering drugs, analgesic drugs) were excluded. Therefore, it is suggested that this relationship should be considered in the follow-up phase of this prospective study. Also, since our research was a cross-sectional descriptive study, the causal relationship between the variables should be further investigated using longitudinal studies.

## Data Availability

The datasets used and/or analysed during the current study are available from the corresponding author on reasonable request.

## Abbreviations

ALP: Alkaline phosphatase
ALT: Alanine aminotransferase
AST: Aspartate aminotransferase
